# Vitamin D and airway infections: a European perspective

**DOI:** 10.1186/s40001-016-0208-y

**Published:** 2016-03-24

**Authors:** Armin Zittermann, Stefan Pilz, Harald Hoffmann, Winfried März

**Affiliations:** Department of Thoracic and Cardiovascular Surgery, NRW Heart and Diabetes Centre, Clinic for Thoracic and Cardiovascular Surgery, Ruhr University of Bochum, Georgstraße 11, 32545 Bad Oeynhausen, Germany; Department of Endocrinology and Metabolism, Medical University of Graz, Graz, Austria; Synlab MVZ Gauting, Institute of Microbiology and Laboratory Medicine, WHO Supranational Reference Laboratory of Tuberculosis, Gauting, Germany; Synlab Academy for Continuing Medical Education, Mannheim und Synlab Services GmbH, Augsburg, Germany; Clinical Institute for Medical and Chemical Laboratory Diagnostics, Medical University of Graz, Graz, Austria; Department of Medicine V (Nephrology, Hypertension, Rheumatology, Endocrinology, Diabetology) Mannheim Faculty of Medicine, University of Heidelberg, Heidelberg, Germany

**Keywords:** Vitamin D, 25-hydroxyvitamin D, Infection, Immune defence, Tuberculosis, Acute airway infection

## Abstract

Vitamin D has immuno-modulatory properties, and deficient levels of circulating 25-hydroxyvitamin D (<30 nmol/l) may contribute to increased risk of infectious illnesses. This narrative review summarises data on vitamin D status in Europe and updates results of randomised controlled trials (RCTs) regarding vitamin D and airway infections such as tuberculosis (TB) and acute upper respiratory tract infection. In Europe, the prevalence of vitamin D deficiency is up to 37 % in the general population and up to 80 % in nursing home residents and non-European immigrants. Half of TB patients have a migration background. While results of RCTs do not support the concept of beneficial adjunctive effects of vitamin D supplements in anti-TB treatment [odds ratio (OR) = 0.86; 95 % CI 0.62–1.19], the few published RCTs on the prophylaxis of TB suggest some protective vitamin D effects in individuals with deficient circulating 25-hydroxyvitamin D levels. Regarding acute respiratory tract infection, RCTs indicate a significant risk reduction by vitamin D supplements [OR = 0.65; 95 % confidence interval (CI) 0.50–0.85]. There is evidence that daily administration is more effective than high-dose bolus administration [OR = 0.48 (95 % CI 0.30–0.77) vs. OR = 0.87 (95 % CI 0.67–1.14)] and that individuals with deficient or insufficient (30–50 nmol/l) circulating 25-hydroxyvitamin D levels benefit most. Several vitamin D effects on innate immunity may explain these protective effects. In summary, there is possible evidence from RCTs for protective vitamin D effects on TB and likely evidence for protective effects on acute airway infection. Since vitamin D deficiency is prevalent in Europe, especially in institutionalised individuals and non-European immigrants, daily oral vitamin D intake, e.g. 1000 international units, is an inexpensive measure to ensure adequate vitamin D status in individuals at risk.

## Background

Acute respiratory tract illnesses are very prevalent. Most people will develop an infection every year. The majority of these infections are caused by viruses, but suppurative and non-suppurative bacterial complications are also possible [1]. Common cold and influenza are frequent upper respiratory tract infections (URTIs) [2]. Pneumonia is a lower respiratory tract infection, which tends to be a far more serious condition than URTIs, such as common cold [3]. Airway infections are an important cause of disability, days lost from school or work, hospitalisation and mortality [4–6]. In temperate regions like Europe, there is strong seasonality of airway infections, with peak levels being observed in the winter [7]. Tuberculosis (TB), caused by the intracellular pathogen *M. tuberculosis* (MTB), is an infection of the lower respiratory tract. In approximately 80 % of cases, TB affects the lung, and in 20 %, it affects any extrapulmonary organ of the body including the skin. In Europe, tuberculosis was widespread in the 19th and early 20th centuries and was associated with high mortality rates [8]. TB is still among the worldwide number one killers among infectious diseases caused by single pathogens, with more than 9 m new cases and more than 1.5 m deaths per year [9].

It has been assumed that vitamin D status may influence infectious diseases like TB and acute URTI (see below). This assumption is based on findings that vitamin D has important effects on the immune system (see below). This narrative review provides data on vitamin D status in Europe, the history of TB treatment by vitamin D in Europe, and updates results of randomised controlled trials (RCTs) on vitamin D and airway infections such as TB and acute URTI.

## Vitamin D metabolism and status classification

**Fig. 1:**
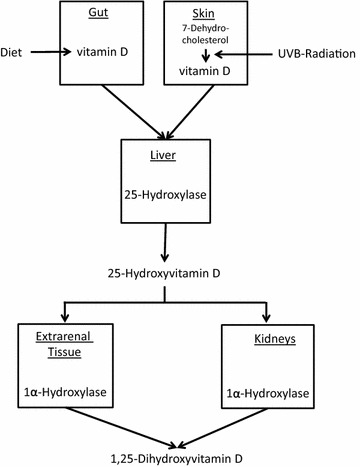
Simplified schematic vitamin D metabolism. Dietary vitamin D and endogenously produced vitamin D are both first metabolised in the liver into 25-hydroxyvitamin D and then in the kidney and various extra-renal tissues into the vitamin D hormone 1,25-dihydroxyvitamin D

Vitamin D has a special position among the vitamins because the human body is able to synthesise it from the precursor molecule 7-dehydrocholesterol (Fig. 1). This is generated by the skin upon sufficient exposure to solar ultraviolet radiation in wavelengths from 290 to 315 nm (UVB radiation). Measurable quantities of vitamin D also occur in some natural foods, in particular in oily fish like eel, salmon and herring, as well as in cod liver oil. Since its final effector molecule is produced in the human organism, some authors consider vitamin D more a pro-hormone than a vitamin sensu stricto [10]. With modern lifestyles, however, the majority of human beings worldwide stay indoors for most of the time. This certainly leads to diminished or even absent synthesis of vitamin D in the skin, in particular in certain risk groups including office workers, nursing home residents, coloured populations living in northern latitudes and women wearing traditional dresses which cover the whole body. Due to increasing numbers of people falling into one of the aforementioned risk groups, it is very likely that in future the vitamin nature of this essential substance will become the focus of interest, i.e. the need for its oral intake.

Vitamin D status is best determined by measuring circulating 25-hydroxyvitamin D (25OHD) in plasma or serum. 25OHD is the first hydroxylation product of vitamin D and is synthesised in the liver. The North American Institute of Medicine (IOM) has classified serum 25OHD levels of 50 to 125 nmol/l [20 to 50 ng/ml] as sufficient [1 nmol/l = 0.4 ng/ml], levels between 30 and 49.99 [12 and 19.99 ng/ml] as inadequate, levels below 30 nmol/l [12 ng/ml] as deficient and levels above 125 nmol/l [50 ng/ml] as potentially harmful [11]. The ‘deficient’ and ‘inadequate’ categories have been set up exclusively based on the effects of vitamin D on bone health. The assumption of potentially harmful vitamin D effects at levels above 125 nmol/L originates from results of prospective cohort studies of tumour incidence, myocardial infarction and total mortality [11]. The Endocrine Society (ES), in contrast, has set the lower threshold of adequate 25OHD concentrations to 75 nmol/l (30 ng/ml), i.e. 50 % higher than the IOM [12]. The ES-recommendation is not only based on convincing evidence of positive vitamin D effects on the musculoskeletal system but also on potential evidence of positive extra-skeletal effects. The ES did not, however, define an upper threshold of 25OHD levels.

The metabolically active form is 1,25-dihydroxyvitamin D (1,25[OH]_2_D), which is produced from 25OHD mainly in the kidney but also in different extra-renal tissues. The human organism usually regulates serum concentration of 1,25(OH)_2_D between quite strict limits. Therefore, 1,25(OH)_2_D levels are clinically less meaningful. However, once the vitamin D supply becomes severely deficient, the circulating 1,25(OH)_2_D level becomes substrate dependent, i.e. dependent on circulating 25OHD levels, and secondarily decreases as well [13].

To guarantee adequate vitamin D intake, the IOM recommends daily vitamin D intakes of 400, 600 and 800 international units (1 IU = 0.025 µg) in the first year of life, following infancy up to 70 years of age, and beyond 70 years of age, respectively. This corresponds well with the recommendations of the German, Austrian and Swiss Nutrition Society (D-A-CH) [11, 14]. IOM and European Food Safety Authority have set 4000 IU as the maximum permissible dose of safe daily vitamin D intake.

## Literature review strategy and methods

We performed a systematic literature search in PubMed and Google Scholar without language restrictions for relevant publications released until June 30th, 2015, using the following terms: (“vitamin D” or “cholecalciferol” or “25-hydroxyvitamin D”) and (“immune system” or “infection” or “infectious disorder” “infectious disease” or “immune defence” or “influenza” or “upper respiratory tract infection” or “tuberculosis” or “airway infection”). Personal collections of articles on this topic as well as references from selected articles were also used to extend the search. Some articles are not cited due to space limitations. We systematically analysed retrieved RCTs on vitamin D and tuberculosis/airway infection in an approach similar to meta-analyses. We used RevMan (Review Manager. Version 5.3.: The Nordic Cochrane Centre. The Cochrane Collaboration. Copenhagen, 2014) to perform the analyses.

## Epidemiology of vitamin D deficiency in Europe

Circulating 25OHD levels can vary widely according to lifestyles, skin pigmentation, dietary vitamin D intake, season, latitude and health status. In the general adult population of selected European countries (Austria, Germany, United Kingdom, Denmark, Finland, Ireland and Poland), the prevalence of vitamin D deficiency, e.g. 25OHD levels <25 nmol/l, is 11.6–37 % [15]. Moreover, the HELENA (healthy lifestyle in Europe by nutrition in adolescence) study has demonstrated that 15 % of European adolescents have 25OHD levels <27.5 nmol/l [16]. This figure is similar to results of a representative German survey in children and adolescents, where approximately 16 % had 25OHD levels <25 nmol/l [17]. Usually, peak levels of circulating 25OHD are achieved in summer, whereas a nadir is observed at the end of the winter. Results from a recent retrospective survey with 98,000 patients tested between 2008 and 2011 in Northern Germany support the season dependency of vitamin D deficiency. Less than 10 % and near to 40 % of participants had vitamin D deficiency during the summer and the winter seasons, respectively [18]. In a nationwide cohort of British adults at age 45 years, the prevalence of 25OHD levels <25 nmol/l was 3.2 % in summer/autumn and 15.5 % in winter/spring [19]. That study also demonstrated latitudinal differences in 25OHD levels, with 8 % higher prevalence of deficient 25OHD levels in Scotland compared with the south of England. Data are in general agreement with a French study reporting in middle-aged men and women with a mean 25OHD level of 43 nmol/l in Northern France and 94 nmol/l in the southwest of France [20]. A particular risk group for vitamin D deficiency is non-European immigrants. The aforementioned German survey reported deficient 25OHD levels in up to 30 % of migrant girls, compared with 17 % of native German girls [17]. In the Netherlands and Denmark, serum 25OHD was below 25 nmol/l in up to 80 % of non-European immigrants, with particularly high prevalence in girls and women [15]. Another particular risk group for vitamin D deficiency is frail elderly people. In recent studies from Germany and Austria [21, 22], the supply situation was particularly poor in rehabilitation patients and nursing home residents. 67 and 75 % had vitamin D deficiency, respectively. In view of these devastating vitamin D deficiency statistics, it should be taken into consideration that the automated immunoassays measuring 25OHD levels in blood may markedly vary, with significant impact on vitamin D status classification [23]. In November 2010, an international collaborative initiative organised by the office of dietary supplements of the National Institutes of Health therefore established a vitamin D standardisation program (VDSP) [24]. A recent study applied VDSP protocols to serum data from representative childhood/teenage and adult/older adult European populations [25]. An overall pooled estimate, irrespective of age, showed that 13 % of the 55,844 European individuals had yearly mean standardised serum 25OHD levels <30 nmol/l. In Finnish and British immigrant groups, the prevalence was between 28 and 50 %.

## History of treatment of airway infection

Cod liver oil and UVB radiation, which today are known to be the most effective sources of vitamin D for humans, have long been used to treat airway infections such as tuberculosis in Western Europe [26, 27]. In 1848, in one of the very first clinical trials on the treatment of TB, more than 1000 TB patients were either assigned to the verum group and received cod liver oil three times daily, or to the control group with simple nursing care. 33 % of the control group versus 19 % of the verum group experienced significant worsening of their disease or died [26]. In 1903, Niels Finsen was awarded the Nobel Prize in Physiology or Medicine for the proof that phototherapy can cure lupus vulgaris (skin tuberculosis) [28]. In the absence of effective antibiotics and chemotherapy, heliotherapy (helios = sun) was quite popular in Western Europe, exposing TB patients to UV rich sunlight in high altitudes [27]. After the discovery of synthetic antituberculous substances, starting with streptomycin in the 1940s, heliotherapy was displaced by the more effective antituberculous chemotherapy [29]. In 2006, however, a German-American working group demonstrated that increased expression of the vitamin D receptor and of the 1α-hydroxylase gene (the 1α-hydroxylase synthesises 25OHD) induces synthesis of the antimicrobial peptide cathelicidin in human macrophages [30]. In the same year, Cannell et al. [31] put forward the hypothesis that vitamin D deficiency increases the risk of contracting influenza in winter. Of note, during recent years, several other natural products such as bacterial lysates or components of bacterial cells (ribosomal extracts), prebiotic oligosaccharides, probiotics and yeast-derived beta-glucans have also been successfully used in airway infection prevention [32–35].

## Clinical associations of vitamin D with tuberculosis

Two effects have so far assigned to vitamin D with regards to TB: (i) a potentially protective value of sufficient vitamin D levels against reactivation of TB infections and (ii) the therapeutic effect of vitamin D supplementation on the clinical outcome of the active disease. With respect to preventive vitamin D effects, it is noteworthy that in European countries, approximately half of TB patients have a migration background. Globally, the prevalence of TB per 100,000 people is highest in sub-Saharan Africa and is also relatively high in Asia. It has been argued that immigrants become infected in their countries of origin and that the infection reactivates upon enhanced vitamin D deficiency due to weaker sunlight in the Northern regions [36]. So far, two RCTs [37, 38] have investigated the protective effect of vitamin D on TB. One trial [37] daily supplemented a verum group of school children in the high TB incidence setting of Mongolia with 800 IU and compared the additional numbers of latent TB infections measured with a tuberculin skin test with a placebo control group. The authors found a strong protective trend in the verum group (risk ratio: 0.41; 95 CI 0.16–1.09). Unfortunately, they did not provide baseline data on smear-positive pulmonary TB. The other trial [38] investigated the effect of a single dose of 100,000 IU vitamin D on adult persons who had contact to TB index cases in the United Kingdom (UK). The authors could not see a protective effect on the risk of acquiring a latent TB infection as measured using ESAT-6/CFP-10-based interferon-gamma release assays. The authors observed, however, an enhanced antimicrobial in vitro activity of blood from verum group patients compared to controls against bacteria of a BCG vaccine strain. But the clinical relevance of this finding could not yet be established. Notably, in the Mongolian study, initial 25OHD levels were on average below 18 nmol/l, and thus in the range, the IOM has classified as deficient. In the UK study, mean initial 25OHD levels were 35 nmol/l and thus slightly above the deficiency range.

**Fig. 2:**
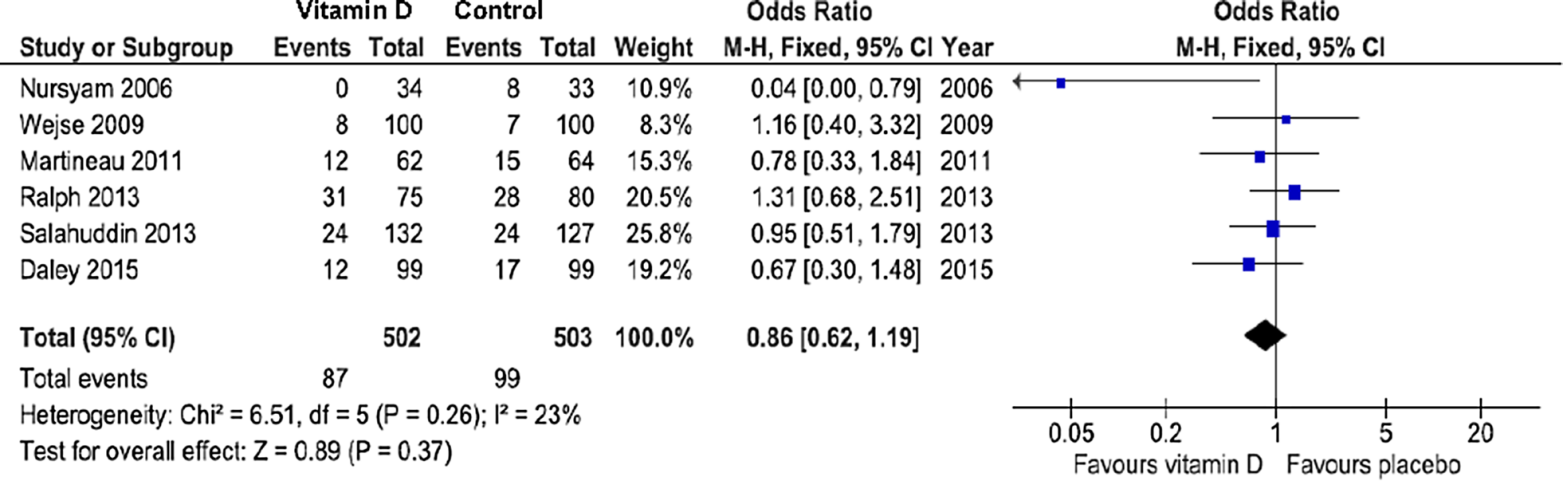
Meta-analysis of the efficacy of vitamin D therapy against tuberculosis. Results are presented as odds ratios. *Error bars* indicate 95 % confidence intervals

Table 1 lists six published RCTs [39–44] which have examined the therapeutic effect of vitamin D on the course of the disease. Four studies, one study and one study were conducted in Asia, Africa, and the UK, respectively. Different forms of pulmonary and extrapulmonary TB were included in the studies. In five of the six trials, high bolus doses of vitamin D were administered once, on a weekly or on a monthly basis. One study only had daily supplemented vitamin D at a dose of 10,000 IU. In two studies, 25OHD levels were not measured, and in three other studies, initial 25OHD levels were on average already above 50 nmol/l. Results of individual studies and summary results are illustrated as odds ratios (ORs) and 95 % confidence intervals (CI) in Fig. 2. Cases which did not terminate the treatment according to the respective study protocols had to be excluded. Since there was no evidence of heterogeneity in study results (*I*^2^ = 23 %; *P* = 0.26), we used a fixed effects model. Overall, there was a non-significant reduction of TB infections in the vitamin D group (odds ratio: 0.86; 95 % CI 0.62–1.19; *P* = 0.338). Although in the study by Martineau [41] vitamin D did not significantly affect time to sputum culture conversion in the whole study population, it did significantly hasten sputum culture conversion in participants with the tt genotype of the TaqI vitamin D receptor polymorphism, indicating that vitamin D availability in target cells may indeed influence disease outcome. The studies were not designed to answer the question of whether vitamin D was able to reduce mortality in TB patients. Results would have been of scientific interest since earlier systematic reviews and meta-analyses of RCTs had already demonstrated that vitamin D supplements reduce mortality from a variety of diseases [45, 46]. Interestingly enough, several studies have also associated vitamin D deficiency with other forms of lower respiratory tract infections [47–50].

**Table 1:** Randomised, controlled trials of vitamin D for treatment of active tuberculosis

Author (ref.)	Publication year	N (total)	Age (years)	Initial 25OHD (nmol/l)		In-study 25OHD (nmol/l)		Mean vitamin D dose (IU)	Duration	Endpoint
				Vit D	Placebo	Vit D	Placebo			
Nursyam [39]	2006	67	31	ND	ND	ND	ND	10,000 daily	6 weeks	Sputum conversion
Wejse [40]	2009	365	37	77	79	98	95	100,000 quarterly	12 months	Sputum conversion
Martineau [41]	2011	146	31	21	21	101	23	4 × 100,000	6 days	Sputum conversion
Salahuddin [42]	2013	259	28	52	58	220	50	2 × 600,000	12 weeks	Sputum conversion
Ralph [43]	2013	200	28	ND	ND	ND	ND	50,000 monthly	8 weeks	Sputum conversion
Daley [44]	2015	247	43	58	54	72	60	100,000 quarterly	12 months	Sputum conversion

*25OHD* 25-hydroxyvitamin D; *IU* international units; *ND* not determined

## Clinical associations of vitamin D with acute airway infections

**Fig. 3:**
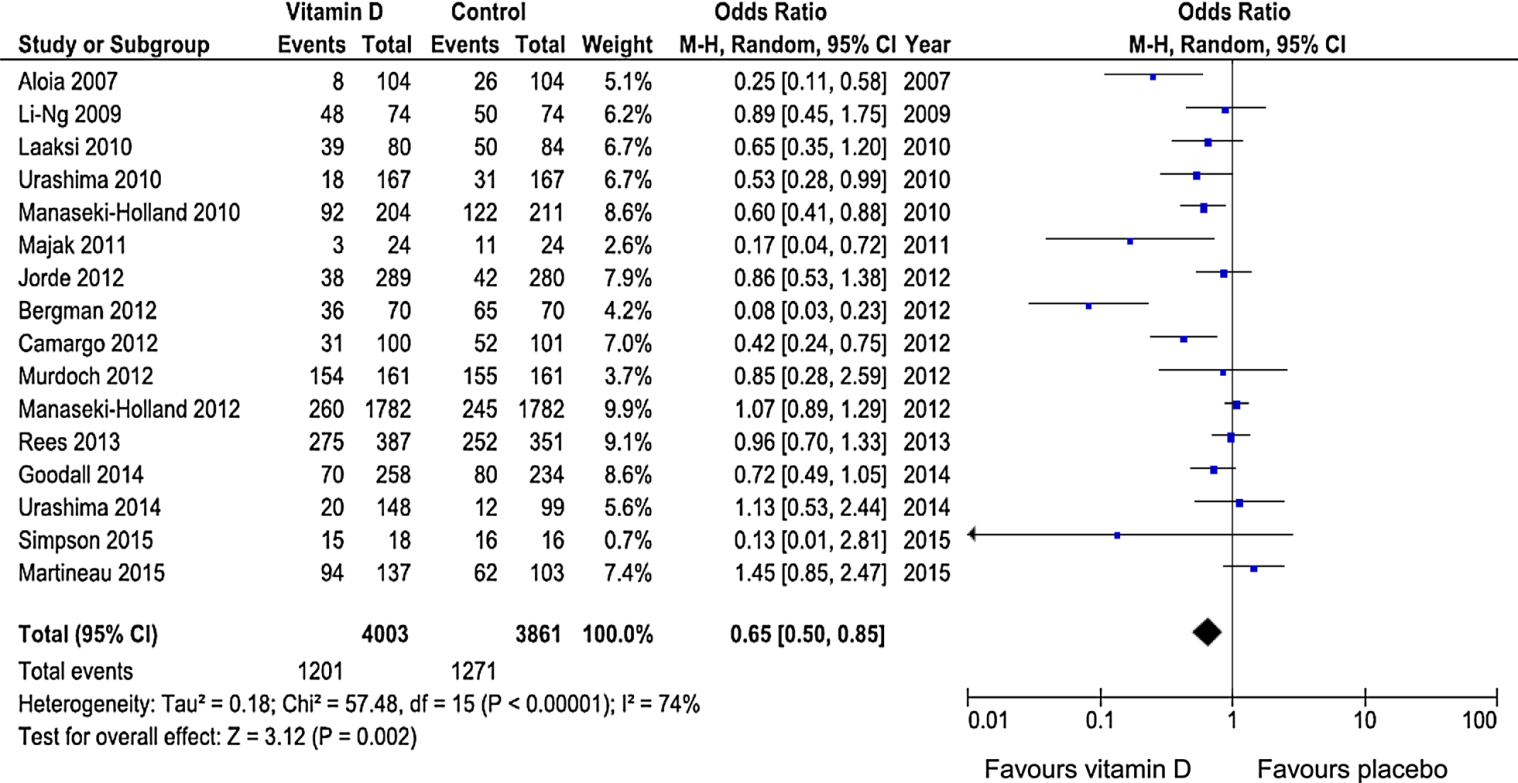
Meta-analysis of the efficacy of vitamin D therapy against acute airway infections. Results are presented as odds ratios. *Error bars* indicate 95 % confidence intervals

Studies of the influence of vitamin D on URTIs are methodically difficult because the duration and extent of the infections are not always easy to record objectively. Against this background, a Finnish study [51] stands out: absence from duty due to respiratory tract infections was studied in 800 recruits, i.e. a homogeneous group of fundamentally healthy young men. It showed that recruits with 25OHD concentrations of <40 nmol/l (16 ng/ml) were unfit for work significantly more frequently than recruits with higher 25OHD. Based on these data, a randomised controlled extension trial, in which the recruits received either 400 IU of vitamin D or a placebo, was conducted over the winter season (October to March) [52]. The mean duration of days of absence from duty tended to be lower in the vitamin D group than in the placebo group (on average 2.2 vs. 3.0 days). Furthermore, the percentage of recruits with absence was significantly lower than in the placebo group (35.7 vs. 51.3 %). A meta-analysis of RCTs published by Bergman et al. [53] in 2013 systematically analysed the influence of vitamin D intake on the risk of respiratory tract infection [53]. Included studies [52, 54–63] are listed in Table 2. Since publication of this meta-analysis [53], several other RCTs [64–68] have been published on this topic and are presented in Table 2, resulting in 16 studies in total. Of these 16 studies, 11 were conducted in healthy subjects and five in patients, the study participants being children in six studies and adults in eleven studies. All 16 studies were included in a new systematic analysis using a random effect model (Fig. 3). Outcome was the number of patients experiencing at least one episode of infection. Overall, vitamin D administration reduced the risk of infection significantly (odds ratio = 0.65; 95 % CI 0.50–0.85; *P* = 0.005). Exclusion of the two studies by Manaseki-Holland et al. [56, 62] that used vitamin D to protect against a (repeat) episode of pneumonia did not change results substantially (odds ratio = 0.61; 95 % CI 0.44–0.84; *P* < 0.001). However, results showed significant heterogeneity among studies (*I*^2^ = 74 %, *P* < 0.001), supporting the need for a random effects model. Subgroup analysis indicated that daily vitamin D administration was associated with a better outcome than (high-dose) bolus vitamin D administration [odds ratio = 0.48 (95 % CI 0.30–0.77) vs. odds ratio = 0.87 (95 % CI 0.67–1.14)]. Moreover, in three trials with initial 25OHD levels <50 nmol/l [54, 60, 68], the odds ratio of vitamin D effectiveness was 0.55 (95 % CI 0.20–1.55), indicating that initial 25OHD level may also influence vitamin D effectiveness. Notably, Simpson et al. [67] reported that a protective vitamin D effect could only be observed in the subgroup of individuals with initial 25OHD levels <40 nmol/l. In these individuals, vitamin D resulted in a 44 % reduction in infection risk (*P* = 0.007). URTI and non-URTI were included in this analysis. In the study by Rees et al. [64], a further observational analysis of their data suggested a significant decreased rate ratio of 0.91 (95 % CI 0.83–0.99) for URTI per 25 nmol/l increase in 25OHD. A few years ago, the analysis of an extensive US data set [69] revealed, as expected, that URTI shows strong seasonal variation, with the frequency peaking in winter. However, people with deficient circulating 25OHD (<25 nmo/l or <10 ng/ml) always exhibited a higher risk than people with adequate circulating 25OHD (>75 nmol/l or >30 ng/ml).

**Table 2:** Randomised, controlled trials of vitamin D for prevention of respiratory tract infections

Author (ref.)	Publication year	N (total)	Age (years)	25OHD (nmol/l)		25OHD (nmol/l)		Mean vitamin D dose (IU)	Duration	Endpoint
				Initial		In-study				
				Vit D	Placebo	Vit D	Placebo			
Aloia [54]	2007	208	60.6	46.9	43	86.9	43	800/2000 daily	3 years	Influenza
Li-Ng [55]	2009	148	58.7	58.7	63	88.5	60.9	2000 daily	3 months	URTI
Laaksi [52]	2010	164	Young men	78.7	74.4	72	51	400 daily	6 months	Acute RTI
Manaseki-Holland [56]	2010	453	1.2	ND	ND	ND	ND	100,000 (single dose)	3 weeks	Pneumonia
Urashima [57]	2010	334	10.2	ND	ND	ND	ND	1200 daily	4 months	Influenza A
Majak [58]	2011	48	11.5	64.3	88	94	80	500 daily	6 months	Acute RTI
Bergman [59]	2012	124	53.1	51.5	46.9	117.4	44	4000 daily	12 months	RTI
Camargo [60]	2012	244	19.9	17.5	17	47.3	18	300 daily	7 weeks	Acute RTI
Jorde [61]	2012	569	63	ND	ND	ND	ND	3344^c^	12 months	Influenza-like
Manaseki-Holland [62]	2012	3046	0.8	ND	ND	^a^	^b^	100,000 quarterly	18 months	Pneumonia
Murdoch [63]	2012	322	47.5	75.5	70	122.5	55	100,000 monthly^d^	18 months	URTI
Rees [64]	2013	759	58.1	62	63.1	ND	ND	1000 daily	3–5 years	URTI
Urashima [65]	2014	247	Students	ND	ND	ND	ND	2000 daily	2 months	Influenza A
Goodall [66]	2014	471	19	ND	ND	ND	ND	10,000 weekly	2 months	URTI
Simpson [67]	2015	34	Healthy adults	60.5	76.4	107	46	20,000 weekly	17 weeks	RTI
Martineau [68]	2015	250	47.9	49.8	49.4	69.4	46.5	120,000 bimonthly	1 year	Acute RTI

*25OHD* 25-hydroxyvitamin D; *IU* international units; *ND* not determined; *URTI* upper respiratory tract infection; *RTI* respiratory tract infection

^a^significantly higher than placebo

^b^significantly lower than vitamin D group

^c^several studies were included. The vitamin D doses in the individual studies were as follows: 2000, 2800 and 6800 IU/daily, 20,000 and 40,000 IU/weekly and 100,000/monthly, every 2 months or every 3 months

^d^ initial dose: 200,000 IU

The results on acute respiratory tract infection are particularly interesting for two reasons: firstly, the data prove that vitamin D, in addition to its effects on the musculoskeletal system, also possesses other important functions of clinical relevance. Secondly, the data show that attention must be paid to the vitamin D supply not only during infancy and old age but also in otherwise healthy children and adults. Notably, the winter nadir in vitamin D status parallels the winter peak in airway infections.

As viral infections of the respiratory tract often precede bacterial infections, the results of a post hoc analysis of the RECORD trial [70], a prospective randomised study with 5300 participants who received either 800 IU of vitamin D or a placebo daily for two to 5 years, are interesting: in the vitamin D group, there was a tendency towards fewer self-reported infections (of any cause) and antibiotics use. Results are in line with data by Bergman et al. on respiratory tract infection [59], demonstrating a 63.5 % reduction in the taking of antibiotics in the vitamin D group. In another post hoc analysis of a vitamin D study, an age-related effect of vitamin D on antibiotic intake was observed [71]: while vitamin D supplements did not reduce antibiotic intake in people under 70 years of age, patients aged 70 years and older in the control group required considerably more antibiotics than those in the vitamin D group. Although the results of the aforementioned three studies are provisional, and further studies on reduction of the need for antibiotics due to vitamin D are necessary, they provide promising evidence of a role of protective vitamin D effects on respiratory tract infections. Altogether, likely evidence exists that a sufficient vitamin D supply can prevent URTI.

## Molecular effects of vitamin D on the immune system

Molecular vitamin D effects on the immune system may explain the protective clinical associations observed (Table 3). Since the 1980s, it has become increasingly clear that vitamin D plays a prominent role in innate immunity. Vitamin D receptors are found on monocytes. These cells differentiate into macrophages under the influence of 1,25(OH)_2_D [72]. Macrophages express their own 1α-hydroxylase isoenzyme which intracellularly synthesises the active 1,25(OH)_2_D. The activity of this enzyme is increased in activated macrophages [73]. During phagocytosis, macrophages incorporate pathogens such as MTB in the so-called phagosomes. Human macrophages which are infected with MTB show higher expression of the intracellular vitamin D receptor and of the 1α-hydroxylase in human macrophages than native macrophages [30]. 1,25(OH)_2_D directly and indirectly regulates the expression of cathelicidin and defensins, respectively [28, 30]. Cathelicidin induces the fusion of inactive phagosomes with active autophagosomes [74]. Under the further influence of cathelicidin, the autophagosomes can fuse with lysosomes to the autolysosome [75]. As the next weapon of innate immunity, 1,25(OH)_2_D induces the expression of lysosomal enzymes and of reactive oxygen species like nitric oxide, ultimately leading to increased antimicrobial activity [75]. The combination of antimicrobial peptides and oxygen species may destroy intracellular viruses, fungi, and bacteria in the autolysosomes. Due to cathelicidin’s antiviral effects, it supports host defence against influenza virus or human immunodeficiency virus [76]. Notably, monocytic cathelicidin production is reduced in individuals with insufficient 25OHD or low 1,25(OH)_2_D levels [77].

**Table 3:** Vitamin D effects on innate and adaptive immunity

Innate immunity	• Cell types: Monocytes/macrophages, keratinocytes, gastrointestinal/bronchial epithelial cells, decidual cells, trophoblastic cells, natural killer cells • Vitamin D effects: Antimicrobial peptides (cathelicidin, defensins)↑ Autophagy↑ Reactive oxygen species↑, Nitric oxide↑ Cytokines (e.g. interleukins 6, 8, 12, tumour necrosis factor-α)↑ Chemokines (e.g. CCL3, CCL4, CCL8, CCL20)↑
Adaptive immunity	• Cell types: T cells, dendritic cells • Vitamin D effects: T-regulatory cells↑, T helper cells type 17↓ T helper cells type 1↓, T helper cells type 2↑ Pro-inflammatory cytokines (e.g. interleukin 6, tumour necrosis factor-α)↓ Anti-inflammatory cytokines (e.g. interleukins 4 and 10)↑ Immunoglobulin E↑

↑ denotes increase

↓ denotes decrease

The vitamin D innate host response is also active in many other cells like keratinocytes, gastrointestinal/bronchial epithelial cells, decidual cells, trophoblastic cells and natural killer cells [76, 78]. Accordingly, vitamin D bacterial action is broad-reaching and can provide protection against various pathogens, especially in airway infections [76]. By inducing the expression of defensins, vitamin D seems indirectly to help blocking of the membrane fusion mediated by viral hemagglutinin, which might partly explain the antiviral potential that is ascribed to vitamin D, particularly with regard to influenza [57]. Finally, vitamin D plays a role in acquired immunity directly by acting on T cells and indirectly by regulating dendritic cells [76]. Vitamin D-mediated induction of immunoglobulin E expression together with eosinophil granulocytes supports the elimination of some extracellular pathogens like parasites or fungi [79]. Vitamin D also restricts Th1/Th17 cell differentiation and favours Th2 differentiation [76]. High vitamin D levels seem to be concomitant with decreased pro-inflammatory cytokines, which might have a positive effect on disease progression [57, 80]. Conversely, a low vitamin D status is associated with an activation of inflammatory processes [81]. Since it takes approximately 48 h between antigen recognition and full T cell activation, it has been speculated that if the innate immune system is able to clear the infection rapidly, the vitamin D-mediated delay of full T cell activation puts the brakes on excessive T cell proliferation to avoid immunopathology [76].

## Conclusion

Vitamin D is an essential substance for the human body, but large population-based studies demonstrate that vitamin D deficiency is widespread in Europe. Institutionalised individuals and non-European immigrants are most affected. Moreover, the proportion of the latter group in European TB cases is high. Therefore, non-European immigrants would probably benefit from improving their vitamin D status. Present data indicate that vitamin D deficiency increases the risk of acute airway infection. Of note, there is also evidence that vitamin D can lower the risk of other common infections like sepsis, human immunodeficiency virus and hepatitis C virus [76, 82–86]. Due to the available evidence, the high prevalence of deficient vitamin D status in the general European population, and taking into consideration the benefit/risk ratio, vitamin D supplementation can therefore be considered for infection prophylaxis. Oral vitamin D needs are increased, particularly in winter, due to the lower solar UVB radiation at this time of the year. Recommended oral vitamin D intakes of different nutrition, osteoporosis and endocrine societies vary between 600 and 2000 IU daily [10, 11, 14, 87, 88]. However, so far, recommendations have primarily been based on musculoskeletal health and mortality. Regardless of whether airway infection is considered, there is general agreement that the lower target circulating 25OHD should be 50 nmol/l. From a recently published systematic review of RCTs, it can be concluded that, in the majority of people, daily intake of 1000 IU should result in circulating 25OHD levels >50 nmol/l [89]. Thus, it is reasonable and safe to take approximately 1000 IU of vitamin D daily to optimise non-specific immunity and prevent infection. When doing so, it is important to start supplementation in early autumn in order to ensure an adequate vitamin D level in winter. Supplementation should also take place all year round in people with an increased risk of vitamin D deficiency like office workers, non-European immigrants and frail elderly people. Vitamin D supplements are inexpensive (approx. 6,−€/100 tablets = 22,−€/year) and have the advantage that they target not only the immune system but also other tissues like the musculoskeletal system.

## References

[1] National institute of health and care excellence. Respiratory tract infections—antibiotic prescribing. NICE clinical guideline 69. Guidance.nice.org.uk/cg69. Issued: July 2008.

[2] Zoorob R, Sidani MA, Fremont RD, Kihlberg C (2012). Antibiotic use in acute upper respiratory tract infections. Am Fam Physician.

[3] Faris NS (2014). Respiratory tract bacterial infection. Etiological agents and susceptibility testing. Euro Sci J.

[4] Denny FW (1995). The clinical impact of human respiratory virus infections. Am J Respir Crit Care Med.

[5] Monto AS (2002). Epidemiology of viral respiratory infections. Am J Med.

[6] Monto AS (2004). Occurrence of respiratory virus: time, place and person. Pediatr Infect Dis J.

[7] European Centre for Disease Prevention and Control (2014). Annual epidemiological report 2014—respiratory tract infections.

[8] McCarthy OR (2001). The key to the sanatoria. J R Soc Med.

[9] WHO. Global tuberculosis report 2014. World Health Organization, Geneva, 2014; http://www.who.int/tb/publications/global_report/en/. Accessed 24 Jun 2015.

[10] Vieth R (2004). Why, “vitamin D” is not a hormone, and not a synonym for 1,25-dihydroxy-vitamin D, its analogs or deltanoids. J Steroid Biochem Mol Biol.

[11] Ross AC, Manson JE, Abrams SA, Aloia JF, Brannon PM, Clinton SK (2011). The 2011 report on dietary reference intakes for calcium and vitamin D from the Institute of medicine: what clinicians need to know. J Clin Endocrinol Metab.

[12] Holick MF, Binkley NC, Bischoff-Ferrari HA, Gordon CM, Hanley DA, Heaney RP (2011). Endocrine society. Evaluation, treatment, and prevention of vitamin D deficiency: an Endocrine Society clinical practice guideline. J Clin Endocrinol Metab.

[13] Docio S, Riancho JA, Pérez A, Olmos JM, Amado JA, González-Macías J (1998). Seasonal deficiency of vitamin D in children: a potential target for osteoporosis-preventing strategies?. J Bone Miner Res.

[14] DGE (German Nutrition Society), Österreichische Gesellschaft für Ernährung [Austrian Nutrition Society], Schweizerische Gesellschaft für Ernährungsforschung [Swiss Society for Nutrition Research], Schweizerische Vereinigung für Ernährung [Swiss Nutrition Society]. D-A-CH-Referenzwerte für die Nährstoffzufuhr, 1st edition, 5. revised reprint 2013, Neuer Umschau Buchverlag, Neustadt an der Weinstraße. 2012.

[15] Spiro A, Buttriss JL (2014). Vitamin D: an overview of vitamin D status and intake in Europe. Nutr Bull.

[16] González-Gross M, Valtueña J, Breidenassel C, Moreno LA, Ferrari M, Kersting M, HELENA Study Group (2012). Vitamin D status among adolescents in Europe: the healthy lifestyle in Europe by nutrition in adolescence study. Br J Nutr.

[17] Hintzpeter B, Scheidt-Nave C, Müller MJ, Schenk L, Mensink GB (2008). Higher prevalence of vitamin D deficiency is associated with immigrant background among children and adolescents in Germany. J Nutr.

[18] Kramer J, Diehl A, Lehnert H (2014). Epidemiological study on the dimension of vitamin D deficiency in Northern Germany. Dtsch Med Wochenschr.

[19] Hyppönen E, Power C (2007). Hypovitaminosis D in British adults at age 45 y: nationwide cohort study of dietary and lifestyle predictors. Am J Clin Nutr.

[20] Chapuy MC, Preziosi P, Maamer M, Arnaud S, Galan P, Hercberg S, Meunier PJ (1997). Prevalence of vitamin D insufficiency in an adult normal population. Osteoporos Int.

[21] Schilling S (2012). Epidemic vitamin D deficiency among patients in an elderly care rehabilitation facility. Dtsch Arztebl Int.

[22] Pilz S, Dobnig H, Tomaschitz A, Kienreich K, Meinitzer A, Friedl C (2012). Low 25-hydroxyvitamin D is associated with increased mortality in female nursing home residents. J Clin Endocrinol Metab.

[23] Schmid J, Kienreich K, Gaksch M, Grubler M, Raggam R, Meinitzer A (2013). The importance of assays in vitamin D status classification: a comparison of four automated 25-hydroxyvitamin D immunoassays. Lab Med.

[24] Office of dietary supplements. Vitamin D initiative. https://www.ods.od.nih.gov/Research/VitaminD.aspx. Accessed 30 Jun 2015.

[25] Cashman KD, Dowling KG, Škrabáková Z, Gonzalez-Gross M, Valtueña J, De Henauw S, Moreno L, Damsgaard CT, Michaelsen KF, Mølgaard C, Jorde R, Grimnes G, Moschonis G, Mavrogianni C, Manios Y, Thamm M, Mensink GB, Rabenberg M, Busch MA, Cox L, Meadows S, Goldberg G, Prentice A, Dekker JM, Nijpels G, Pilz S, Swart KM, van Schoor NM, Lips P, Eiriksdottir G, Gudnason V, Cotch MF, Koskinen S, Lamberg-Allardt C, Durazo-Arvizu RA, Sempos CT, Kiely M. Vitamin D deficiency in Europe: pandemic? Am J Clin Nutr. 2016. pii: ajcn120873. [Epub ahead of print]. 10.3945/ajcn.115.120873 PMC5527850 26864360

[26] Kupferschmidt K (2012). Uncertain verdict as vitamin D goes on trial. Science.

[27] Rollier A (1927). Heliotherapy: its therapeutic, prophylactic and social value. Am J Nurs.

[28] Zasloff M (2006). Fighting infections with vitamin D. Nat Med.

[29] Camargo CA, Manson JE (2014). Vitamin D supplementation and risk of infectious disease: no easy answers. Am J Clin Nutr.

[30] Liu PT, Stenger S, Li H, Wenzel L, Tan BH, Krutzik SR (2006). Toll-like receptor triggering of a vitamin D-mediated human antimicrobial response. Science.

[31] Cannell JJ, Vieth R, Umhau JC, Holick MF, Grant WB, Madronich S (2006). Epidemic influenza and vitamin D. Epidemiol Infect.

[32] Arslanoglu S, Moro GE, Schmitt J, Tandoi L, Rizzardi S, Boehm G (2008). Early dietary intervention with a mixture of prebiotic oligosaccharides reduces the incidence of allergic manifestations and infections during the first 2 years of life. J Nutr.

[33] Guillemard E, Tanguy J, Flavigny A, de la Motte S, Schrezenmeir J (2010). Effects of consumption of a fermented dairy product containing the probiotic *Lactobacillus casei* DN-114 001 on common respiratory and gastrointestinal infections in shift workers in a randomized controlled trial. J Am Coll Nutr.

[34] Samuelsen AB, Schrezenmeir J, Knutsen SH (2014). Effects of orally administered yeast-derived beta-glucans: a review. Mol Nutr Food Res.

[35] Kearney SC, Dziekiewicz M, Feleszko W (2015). Immunoregulatory and immunostimulatory responses of bacterial lysates in respiratory infections and asthma. Ann Allergy Asthma Immunol.

[36] Chan TYK (2000). Vitamin D deficiency and susceptibility to tuberculosis. Calcif Tissue Int.

[37] Ganmaa D, Giovannucci E, Bloom BR, Fawzi W, Burr W, Batbaatar D (2012). Vitamin D, tuberculin skin test conversion, and latent tuberculosis in Mongolian school-age children: a randomized, double-blind, placebo-controlled feasibility trial. Am J Clin Nutr.

[38] Martineau AR, Wilkinson RJ, Wilkinson KA, Newton SM, Kampmann B, Hall BM (2007). A single dose of vitamin D enhances immunity to mycobacteria. Am J Respir Crit Care Med.

[39] Nursyam EW, Amin Z, Rumende CM (2006). The effect of vitamin D as supplementary treatment in patients with moderately advanced pulmonary tuberculous lesion. Acta Med Indones.

[40] Wejse C, Gomes VF, Rabna P, Gustafson P, Aaby P, Lisse IM (2009). Vitamin D as supplementary treatment for tuberculosis: a double-blind, randomized, placebo-controlled trial. Am J Respir Crit Care Med.

[41] Martineau AR, Timms PM, Bothamley GH, Hanifa Y, Islam K, Claxton AP (2011). High-dose vitamin D(3) during intensive-phase antimicrobial treatment of pulmonary tuberculosis: a double-blind randomised controlled trial. Lancet.

[42] Salahuddin N, Ali F, Hasan Z, Rao N, Aqeel M, Mahmood F (2013). Vitamin D accelerates clinical recovery from tuberculosis: results of the SUCCINCT Study [supplementary cholecalciferol in recovery from tuberculosis]. A randomized, placebo-controlled, clinical trial of vitamin D supplementation in patients with pulmonary tuberculosis’. BMC Infect Dis.

[43] Ralph AP, Waramori G, Pontororing GJ, Kenangalem E, Wiguna A, Tjitra E (2013). L-arginine and vitamin D adjunctive therapies in pulmonary tuberculosis: a randomised, double-blind, placebo-controlled trial. PLoS One.

[44] Daley P, Jagannathan V, John KR, Sarojini J, Latha A, Vieth R (2015). Adjunctive vitamin D for treatment of active tuberculosis in India: a randomised, double-blind, placebo-controlled trial. Lancet Infect Dis.

[45] Bjelakovic G, Gluud LL, Nikolova D, Whitfield K, Wetterslev J, Simonetti RG (2014). Vitamin D supplementation for prevention of mortality in adults. Cochrane Database Syst Rev.

[46] Chowdhury R, Kunutsor S, Vitezova A, Oliver-Williams C, Chowdhury S, Kiefte-de-Jong JC (2014). Vitamin D and risk of cause specific death: systematic review and meta-analysis of observational cohort and randomised intervention studies. BMJ.

[47] Janssens W, Decramer M, Mathieu C, Korf H (2013). Vitamin D and chronic obstructive pulmonary disease: hype or reality?. Lancet Respir Med.

[48] Larkin A, Lassetter J (2014). Vitamin D deficiency and acute lower respiratory infections in children younger than 5 years: identification and treatment. J Pediatr Health Care.

[49] Rubin BK, Dhand R, Ruppel GL, Branson RD, Hess DR (2011). Respiratory care year in review 2010: part 1. Asthma, COPD, pulmonary function testing, ventilator-associated pneumonia. Respir Care.

[50] Gilbert CR, Arum SM, Smith CM (2009). Vitamin D deficiency and chronic lung disease. Can Respir J.

[51] Laaksi I, Ruohola JP, Tuohimaa P, Auvinen A, Haataja R, Pihlajamäki H (2007). An association of serum vitamin D concentrations <40 nmol/L with acute respiratory tract infection in young Finnish men. Am J Clin Nutr.

[52] Laaksi I, Ruohola JP, Mattila V, Auvinen A, Ylikomi T, Pihlajamäki H (2010). Vitamin D supplementation for the prevention of acute respiratory tract infection: a randomized, double-blinded trial among young Finnish men. J Infect Dis.

[53] Bergman P, Lindh AU, Björkhem-Bergman L, Lindh JD (2013). Vitamin D and respiratory tract infections: a systematic review and meta-analysis of randomized controlled trials. PLoS One.

[54] Aloia JF, Li-Ng M (2007). Epidemic influenza and vitamin D. Epidemiol Infect.

[55] Li-Ng M, Aloia JF, Pollack S, Cunha BA, Mikhail M, Yeh J (2009). A randomized controlled trial of vitamin D3 supplementation for the prevention of symptomatic upper respiratory tract infections. Epidemiol Infect.

[56] Manaseki-Holland S, Qader G, Isaq Masher M, Bruce J, Zulf Mughal M, Chandramohan D (2010). Effects of vitamin D supplementation to children diagnosed with pneumonia in Kabul: a randomised controlled trial. Trop Med Int Health.

[57] Urashima M, Segawa T, Okazaki M, Kurihara M, Wada Y, Ida H (2010). Randomized trial of vitamin D supplementation to prevent seasonal influenza A in schoolchildren. Am J Clin Nutr.

[58] Majak P, Olszowiec-Chlebna M, Smejda K, Stelmach I (2011). Vitamin D supplementation in children may prevent asthma exacerbation triggered by acute respiratory infection. J Allergy Clin Immunol.

[59] Bergman P, Norlin AC, Hansen S, Rekha RS, Agerberth B, Björkhem-Bergman L (2012). Vitamin D3 supplementation in patients with frequent respiratory tract infections: a randomised and double-blind intervention study. BMJ Open.

[60] Camargo CA, Ganmaa D, Frazier AL, Kirchberg FF, Stuart JJ, Kleinman K, Sumberzul N, Rich-Edwards JW (2012). Randomized trial of vitamin D supplementation and risk of acute respiratory infection in Mongolia. Pediatrics.

[61] Jorde R, Witham M, Janssens W, Rolighed L, Borchhardt K, de Boer IH (2012). Vitamin D supplementation did not prevent influenza-like illness as diagnosed retrospectively by questionnaires in subjects participating in randomized clinical trials. Scand J Infect Dis.

[62] Manaseki-Holland S, Maroof Z, Bruce J, Mughal MZ, Masher MI, Bhutta ZA (2012). Effect on the incidence of pneumonia of vitamin D supplementation by quarterly bolus dose to infants in Kabul: a randomised controlled superiority trial. Lancet.

[63] Murdoch DR, Slow S, Chambers ST, Jennings LC, Stewart AW, Priest PC (2012). Effect of vitamin D3 supplementation on upper respiratory tract infections in healthy adults: the VIDARIS randomized controlled trial. JAMA.

[64] Rees JR, Hendricks K, Barry EL, Peacock JL, Mott LA, Sandler RS (2013). Vitamin D3 supplementation and upper respiratory tract infections in a randomized, controlled trial. Clin Infect Dis.

[65] Urashima M, Mezawa H, Noya M, Camargo CA (2014). Effects of vitamin D supplements on influenza A illness during the 2009 H1N1 pandemic: a randomized controlled trial. Food Funct.

[66] Goodall EC, Granados AC, Luinstra K, Pullenayegum E, Coleman BL, Loeb M (2014). Vitamin D3 and gargling for the prevention of upper respiratory tract infections: a randomized controlled trial. BMC Infect Dis.

[67] Simpson S, van der Mei I, Stewart N, Blizzard L, Tettey P, Taylor B (2015). Weekly cholecalciferol supplementation results in significant reductions in infection risk among the vitamin D deficient: results from the CIPRIS pilot RCT. BMC Nutr.

[68] Martineau AR, Hanifa Y, Witt KD, Barnes NC, Hooper RL, Patel M (2015). Double-blind randomised controlled trial of vitamin D3 supplementation for the prevention of acute respiratory infection in older adults and their carers (ViDiFlu). Thorax.

[69] Ginde AA, Mansbach JM, Camargo CA (2009). Association between serum 25-hydroxyvitamin D level and upper respiratory tract infection in the third national health and nutrition examination survey. Arch Intern Med.

[70] Avenell A, Cook JA, Maclennan GS, Macpherson GC (2007). Vitamin D supplementation to prevent infections: a sub-study of a randomised placebo-controlled trial in older people (RECORD trial, ISRCTN 51647438). Age Ageing.

[71] Tran B, Armstrong BK, Ebeling PR (2014). Effect of vitamin D supplementation on antibiotic use: a randomized controlled trial. Am J Clin Nutr.

[72] Provvedini DM, Deftos LJ, Manolagas SC (1986). 1,25-Dihydroxyvitamin D3 promotes in vitro morphologic and enzymatic changes in normal human monocytes consistent with their differentiation into macrophages. Bone.

[73] Zittermann A (2003). Vitamin D in preventive medicine: are we ignoring the evidence?. Br J Nutr.

[74] Jo EK (2010). Innate immunity to mycobacteria: vitamin D and autophagy. Cell Microbiol.

[75] Sly LM, Lopez M, Nauseef WM, Reiner NE (2001). 1alpha, 25-Dihydroxyvitamin D3-induced monocyte antimycobacterial activity is regulated by phosphatidylinositol 3-kinase and mediated by the NADPH-dependent phagocyte oxidase. J Biol Chem.

[76] Kroner Jde C, Sommer A, Fabri M (2015). Vitamin D every day to keep the infection away?. Nutrients.

[77] Dini C, Bianchi A (2012). The potential role of vitamin D for prevention and treatment of tuberculosis and infectious diseases. Ann Ist Super Sanita.

[78] Quesada JM, Serrano I, Borrego F, Martin A, Peña J, Solana R (1995). Calcitriol effect on natural killer cells from hemodialyzed and normal subjects. Calcif Tissue Int.

[79] Kearns MD, Alvarez JA, Seidel N, Tangpricha V (2015). Impact of vitamin D on infectious disease. Am J Med Sci.

[80] Grant WB, Giovannucci E (2009). The possible roles of solar ultraviolet-B radiation and vitamin D in reducing case-fatality rates from the 1918-1919 influenza pandemic in the United States. Dermatoendocrinol.

[81] Murr C, Pilz S, Grammer TB (2012). Vitamin D deficiency parallels inflammation and immune activation, the ludwigshafen risk and cardiovascular health (LURIC) study. Clin Chem Lab Med.

[82] Villar LM, Del Campo JA, Ranchal I, Lampe E, Romero-Gomez M (2013). Association between vitamin D and hepatitis C virus infection: a meta-analysis. World J Gastroenterol.

[83] Watkins RR, Lemonovich TL, Salata RA (2015). An update on the association of vitamin D deficiency with common infectious diseases. Can J Physiol Pharmacol.

[84] Gentile I, Scarano F, Celotti A, DE Iuliis E, Scarano R, Granata V, Pinchera B, Meola M, D’Ambra A, Piccirillo M, DI Paola F, Cavalcanti E, Izzo F, Scarpato N, Borgia G (2015). Low vitamin D levels are associated with the presence of serum cryoglobulins in patients with chronic HCV infection. In Vivo.

[85] Ghosn J, Viard JP (2013). Vitamin D and infectious diseases. Presse Med.

[86] Mansueto P, Seidita A, Vitale G, Gangemi S, Iaria C, Cascio A (2015). Vitamin D deficiency in HIV infection: not only a bone disorder. Biomed Res Int.

[87] Dawson-Hughes B, Mithal A, Bonjour JP, Boonen S, Burckhardt P, Fuleihan GE (2010). IOF position statement: vitamin D recommendations for older adults. Osteoporos Int.

[88] Rizzoli R, Boonen S, Brandi ML, Bruyère O, Cooper C, Kanis JA (2013). Vitamin D supplementation in elderly or postmenopausal women: a 2013 update of the 2008 recommendations from the European society for clinical and economic aspects of osteoporosis and osteoarthritis (ESCEO). Curr Med Res Opin.

[89] Zittermann A, Ernst JB, Gummert JF, Börgermann J (2014). Vitamin D supplementation, body weight and human serum 25-hydroxyvitamin D response: a systematic review. Eur J Nutr.

